# Dynamics of hyperglycemia of patients treated with alpelisib: exploratory interim analysis of ITACA trial

**DOI:** 10.1093/oncolo/oyaf023

**Published:** 2025-03-27

**Authors:** Marija Pancirov, Josipa Flam, Jelena Šuto Pavičić, Dora Čerina Pavlinović, Natalija Dedić Plavetić, Paula Podolski, Žarko Bajić, Mladen Krnić, Eduard Vrdoljak

**Affiliations:** Department of Oncology, University Hospital Center Split, School of Medicine, University of Split, 21 000 Split, Croatia; Department of Radiotherapy and Oncology, University Hospital Center Osijek, School of Medicine, Josip Juraj Strossmayer University of Osijek, 31 000 Osijek, Croatia; Department of Oncology, University Hospital Center Split, School of Medicine, University of Split, 21 000 Split, Croatia; Department of Oncology, University Hospital Center Split, School of Medicine, University of Split, 21 000 Split, Croatia; Department of Oncology, University Hospital Center Zagreb, School of Medicine, University of Zagreb, 10 000 Zagreb, Croatia; Department of Oncology, University Hospital Center Zagreb, School of Medicine, University of Zagreb, 10 000 Zagreb, Croatia; Research Unit “Dr. Mirko Grmek” Psychiatric Clinic Sveti Ivan, 10 000 Zagreb, Croatia; Department of Endocrinology, University Hospital Center Split, School of Medicine, University of Split, 21 000 Split, Croatia

**Keywords:** breast cancer, PIK3CA mutation, hyperglycemia, alpelisib, diet, glucose

## Abstract

**Background:**

Alpelisib and fulvestrant combination has improved outcomes in patients with phosphatidylinositol-4,5-bisphosphate 3-kinase catalytic subunit alpha (PIK3CA)-mutated, hormone receptor-positive (HR+), human epidermal growth factor receptor 2 negative (HER2-)− advanced breast cancer (BC) who relapsed or progressed on prior endocrine therapy. Hyperglycemia, on target toxicity, is a frequent adverse event occurring in over 60% of patients.

**Objectives:**

The ITACA trial explores whether low carbohydrate dietary modifications and evening dosing of alpelisib to potentially mitigate impact of food on hyperglycemia. This exploratory interim analysis aimed to quantify the incidence and timing of hyperglycemia in the ITACA trial’s pooled sample.

**Methods:**

This exploratory interim analysis of the ongoing ITACA trial included 23 patients with HR+, HER2-negative metastatic BC receiving alpelisib and fulvestrant. The exploratory outcomes were grade 2-4 hyperglycemia-free survival and time to onset of hyperglycemia.

**Results:**

Most patients, 21 (91.3%), experienced any-grade hyperglycemia (Grade 1: 9 [39.1%], Grade 2: 8 [34.8%], Grade 3: 4 [17.4%], and Grade 4: 0 [0.0%]) within the first week of alpelisib initiation. The median grade 2-4 hyperglycemia-free survival was 6 days (95% CI 3; 44 days).

**Conclusions:**

This exploratory interim analysis demonstrated the rapid onset of hyperglycemia in patients receiving alpelisib, even with the ITACA trial’s dietary interventions. Proactive monitoring, within the first week after initiation of treatment, and early management of hyperglycemia are crucial in this patient population.

**IMPLICATIONS FOR PRACTICE**

The PIK3CA pathway plays a critical role in maintaining glycemic homeostasis, and its inhibition by alpelisib or other pathway inhibitors can lead to a rapid onset of hyperglycemia in a substantial proportion of patients. Therefore, based on our analysis, it is essential to monitor all patients treated with alpelisib or other PIK3CA pathway inhibitors for hyperglycemia much earlier than the timelines suggested by registration trials—ideally, within the first week of therapy. If feasible, continuous glucose monitoring systems should be used, as these devices can detect glucose abnormalities at the earliest possible stage.

## Introduction

In recent years, female breast cancer (BC) has surpassed lung cancer as the most diagnosed cancer (11.7%) worldwide with ~2.26 million new cases and over 600 000 women deaths in 2020.^1^ Approximately 65% of metastatic BCs are hormone receptor positive (HR+), human epidermal growth factor receptor 2 negative (HER2−).^2^ Endocrine therapy in combination with cyclin-dependent kinase 4 and 6 (CDK4/6) inhibitor is the standard first-line treatment for these patients.^2,3^ The phosphatidylinositol 3-kinase catalytic subunit alpha (*PIK3CA*) mutations occur in ~30%-40% of patients with HR+, HER2 negative BC.^4,5^ Results from the SOLAR-1 trial showed a significant increase in progression-free survival (PFS) with an estimated 35% lower risk of progression or death in patients treated with a combination of alpelisib and fulvestrant that had PIK*3CA*-mutated, HR+, HER2-negative advanced BC who relapsed or progressed on prior endocrine therapy.^6^ The median PFS was 11 months in the alpelisib + fulvestrant group compared with 5.7 months in the fulvestrant alone (HR 0.65).^6^ The BYLieve trial demonstrated effectiveness of alpelisib therapy after progression on a CDK4/6 inhibitor.^7^ These 2 studies placed alpelisib in combination with fulvestrant as second-line treatment of choice after progression to CDK4/6 inhibitors for patients with *PIK3CA*-mutated, HR+, HER2-negative metastatic BC. Unfortunately, many patients treated with alpelisib develop hyperglycemia (in SOLAR-1 trial 63.7% of any grade and 36.6% of Grade 3 or 4) frequently leading to dose reductions, treatment delays, and/or discontinuation.^8^ Hyperglycemia is a reversible, on-target effect of phosphatidylinositol 3-kinase (PI3K) inhibition. It has been observed in both preclinical and clinical studies with alpelisib due to its potential to disrupt glucose and insulin homeostasis.^8,9^

As the phosphatidylinositol 3-kinase/protein kinase B (PI3K/AKT) signaling pathway is responsible for glucose homeostasis, and hyperglycemia is an expected “on-target” side effect because of PI3K inhibition, the most common adverse event in SOLAR-1 was, as expected, hyperglycemia.^6,10^ The median time to onset of Grade ≥ 3 hyperglycemia in SOLAR-1 was 15 days, which is in the base for the summary of product characteristics guidelines for monitoring and management of alpelisib-induced hyperglycemia. Treatment discontinuation was reported in 6.3% and 4.2% of alpelisib + fulvestrant arm due to any grade and grade ≥ 3 hyperglycemia, respectively.^6^

**Figure 1.**
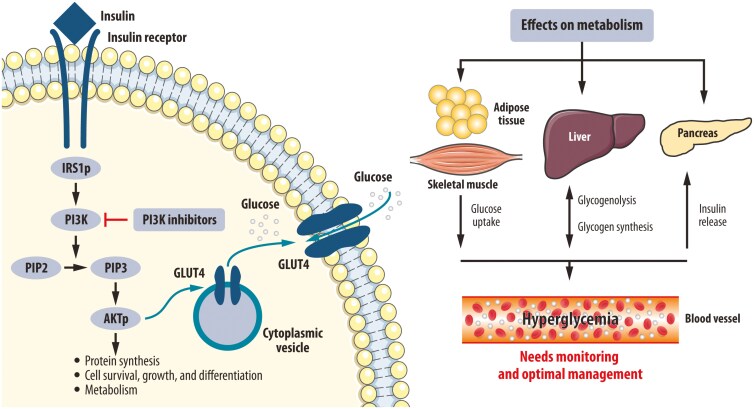
PI3K/AKT pathway inhibition causal path to hyperglycemia. The figure was reproduced with the author’s permission from Tankova et al. 2022.^5^ Abbreviations: AKT, protein kinase B; GLU4, glucose transporter 4; IRS1p, insulin receptor substrate 1, phosphorylated; PI3K, phosphatidylinositol 3-kinase PIP2, phosphatidylinositol 4,5-bisphosphate; PIP3, phosphatidylinositol 3,4,5-trisphosphate.

In preclinical studies, glucose-insulin feedback caused by targeted inhibition of these pathways was sufficient to activate PI3K signaling, even in the presence of PI3K inhibitors. PI3K inhibitors block the production of phosphatidylinositol 3,4,5-trisphosphate (PIP3) and the subsequent activation of AKT. As a result, metabolic disturbances occur, including transient insulin resistance and enhanced glycogenolysis in the liver, which contribute to hyperglycemia and hyperinsulinemia (Figure 1). This insulin feedback could potentially be prevented using dietary or pharmaceutical approaches that increase the efficacy/toxicity ratio of PI3K inhibitors. The rationale for using a ketogenic diet was to deplete hepatic glycogen stores and thereby limit the acute release of glucose from the liver that occurs following PI3K inhibition. While the use of metformin showed limited effect, sodium-glucose co-transporter 2 (SGLT2) inhibitors and the ketogenic diet reduced alpelisib-induced hyperglycemia and consequently release of insulin defining potential ways to further improve toxicity to efficacy ratio.^11-13^

The objective of the ITACA trial (EudraCT number: 2021-000845-42) is to explore the possibility of improving alpelisib efficacy/toxicity ratio by adjusting the patients’ diet and administering alpelisib in the evening (at least 5 hours after the last low-carbohydrate meal) instead of the usual morning dose. Consequently, the potential reduction and/or prevention of hyperglycemia and consequent hyperinsulinemia would potentially increase alpelisib efficacy, decrease incidence of adverse events, and improve the quality of life of our patients. The objective of this explorative interim analysis was to determine timing and the incidence of hyperglycemia in the ITACA trial pooled sample.

## Methods

This is an explorative interim analysis conducted on the pooled sample of the multicentric, randomized, open-label phase IIb ITACA trial currently conducted in 5 Croatian university hospitals. The protocol was approved by the ethics committees of all study centers and registered at European Union Clinical Trials Register (EudraCT) number: 2021-000845-42.

### Trial design and patients’ characteristics

Eligible patients were postmenopausal women or men, ≥18 years of age, with HR+, HER2-negative, *PIK3CA*-mutated metastatic BC with measurable disease or at least one predominantly lytic bone lesion, ECOG performance status 0 or 1, adequate bone marrow and organ function, and progression on previous hormone therapy in combination with CDK4/6 inhibitors. The exclusion criteria, in addition to the other criteria listed in the ITACA trial protocol, was the established diagnosis of diabetes mellitus type 1 or uncontrolled diabetes mellitus type 2. Patients were informed about the trial, received written information, and gave written consent to participate in the study.

### Treatment

Patients were randomized into 2 treatment groups: experimental group (11 patients) who received alpelisib 300 mg orally in the evening daily, at least 5 hours after the last meal which was based on a recommended low carbohydrate diet with a low glycemic index (GI) + fulvestrant 500 mg im monthly (before taking alpelisib, patients eat 200 g of yogurt or 30-50 g of almonds or 100 g of semi-hard cheese with a glass of water) and the control group (12 patients) who received alpelisib 300 mg daily according to European Medicines Agency (EMA) approved recommendations for posology and method of administration + fulvestrant 500 mg im monthly (morning administration of alpelisib without dietary suggestions). Crossover between the 2 groups was not allowed. Treatment with alpelisib was continued until disease progression, assessed by Response evaluation criteria in solid tumors (RECIST) 1.1, unacceptable toxicity, death, or discontinuation from treatment for any other reason. All patients did have 7-point glycemic profile measurements throughout 3 days before therapy initiation as well as 3 days after starting the therapy with alpelisib, followed by twice-daily measurements until the end of alpelisib treatment to optimally describe impact of alpelisib therapy on glucose metabolism for both groups of patients. Patients were randomized in 1:1 ratio using minimization method to balance the following prognostic factors between the 2 arms: age (3 levels: <50; 50-64; ≥65), study center (5 levels), location of metastases (2 levels: visceral metastases vs bone only disease), previous treatment with any CDK4/6 inhibitor (2 levels: yes vs no), previous treatment with chemotherapy (2 levels: yes vs no), glycosylated hemoglobin A1c (2 levels: <5.7% vs* *≥5.7%), treatment with antidiabetic drugs or insulin (2 levels: yes vs no due the fact that previous treatments with insulin or antidiabetic drugs were not explicitly excluded, as patients with controlled diabetes on stable regimens could still participate), body mass index (BMI, <30 kg/m^2^ vs ≥30 kg/m^2^). This exploratory interim analysis was performed on the pooled sample, regardless of randomization.

### Endpoints

The preregistered, original primary endpoint of the ITACA trial is the exposure-adjusted incidence rate of grade 3-4 hyperglycemia within the first 3 months or 30 days after the treatment discontinuation whichever occurs first. The key secondary endpoint was the median time to the first grade 3-4 hyperglycemia from the first dose of study treatment until 30 days from permanent treatment discontinuation. In this exploratory interim analysis of the pooled sample, the key outcome was grade 2-4 hyperglycemia-free survival in days from the alpelisib initiation. We have excluded Grade 1 hyperglycemia from the key outcome measurement due to the fact that 47.8% of our patients had Grade 1 hyperglycemia in pretreatment period (during intensive measurement of 7-point glycemic profile 3 days before taking alpelisib). Other exploratory outcomes of interest were (1) median survival to hyperglycemia of any grade, and (2) of grade 3-4, (3) number of statistically significantly different trends in changes in glucose concentration during the first 14 days after the introduction of alpelisib, (4) the prevalence of any grade, grade 2-4, and grade 3-4 hyperglycemia during the first 7 days, (5) during days 8-14, and (6) during the first 14 days after alpelisib initiation, (7) the median number of days that patient spent in hyperglycemia. In this exploratory interim analysis, hyperglycemia was defined according to Common Terminology Criteria for Adverse Events (CTCAE) version 4.03, as fasting blood glucose (FBG) measured by FreeStyle Libre Sensor in the morning before breakfast: normal blood glucose level: ≤6.1 mmol/L (≤110 mg/dL), grade 1: >6.1-8.9 mmol/L (>110-160 mg/dL), grade 2: >8.9-13.9 mmol/L (>160-250 mg/dL), grade 3: >13.9-27.8 mmol/L (>250-500 mg/dL), and grade 4: >27.8 mmol/L (>500 mg/dL).

### Statistical analysis

We analyzed the key exploratory outcome, median hyperglycemia-free survival, using the Kaplan–Meier method, using product-limit estimators, and generating survival probability curves with 95% CIs. We analyzed trends in changes in glucose concentration during the first 14 days after the introduction of alpelisib using joinpoint regression using the empirical quantiles method for calculation of CI, and the first-order autocorrelated standard errors where autocorrelations were empirically estimated from the data. We selected the optimal model based on the lowest weighted Bayesian information criterion (BIC) and described the detected trends using the average daily percent change (DPC). We calculated prevalence of any-grade, grade 2-4, and grade 3-4 hyperglycemia during the 3 days before and on the last day before the initiation of alpelisib, during the first 7 days, during days 8-14, and during the first 14 days. We controlled the false positive rate using the Benjamini–Hochberg procedure with the false discovery rate set in advance at false discovery rate (FDR) < 5%. We conducted statistical analysis using StataCorp 2019 (Stata Statistical Software: Release 16, College Station, TX: StataCorp LLC) and the joinpoint regression using the Joinpoint Regression Program, version 4.9.1.0-April 2022 (Statistical Methodology and Applications Branch, Surveillance Research Program, National Cancer Institute).

## Results

### Sample characteristics

Between September 17th, 2022 and September 15th, 2023, 23 patients were enrolled and randomly assigned to receive alpelisib in the morning according to the EMA approved recommendation or in the evening according to the ITACA study protocol. For this exploratory interim analysis, data across treatment arms were pooled. The median age of the patients was 63 years (Interquartile range [IQR] 58-70) with median BMI of 26 (IQR 22-30) (Table 1). Most patients had both visceral and bone metastases (74%). Based on the pretreatment laboratory results, median HbA1c at enrolment was 5.6% (IQR 5.2-5.9) and 4 patients (17.4%) had HbA1c higher than 6.1%. During the 3 days before the initiation of alpelisib, 11 (47.8%) patients had FBG > 6.1 mmol/L on at least one measurement, and 5 (21.7%) had it on the last day before beginning of the study treatment (Tables 1 and 2, Supplementary Table S1). All these patients had hyperglycemia of grade 1 at baseline. In this interim analysis, median follow-up from the introduction of alpelisib was 99 (IQR 56-154) days.

**Table 1.** Patients’ characteristics (*n* = 23).

	At baseline
Age at randomization (years), median (IQR)	63 (58-70)
HbA1c (%), median (IQR)	5.6 (5.2-5.9)
HbA1c (%) > 6.1 mmol/l	4 (17)
Body mass index (kg/m^2^), median (IQR)	26 (22-30)
Categorized body mass index (kg/m^2^)	
Normal (<25)	9 (39)
Overweight (25-29.9)	8 (35)
Obese (≥30)	6 (26)
ECOG performance status	
0	17 (74)
1	6 (26)
Location of metastasis	
Visceral	3 (13)
Bone only	3 (13)
Both visceral and bone	17 (74)
Previous chemotherapy	15 (65)
Stage	
I	2 (9)
II	6 (26)
III	7 (30)
IV	8 (35)
Immunophenotype	
Luminal A	8 (40)
Luminal B	12 (60)
Grade	
unknown	6 (26)
1	4 (17)
2	13 (57)
Estrogen receptors, median (IQR)	95 (78-100)
Progesterone receptors, median (IQR)	80 (30-90)
Ki-67, median (IQR)	20 (16-30)
*PIK3CA* mutation	
Unknown	4 (17)
E542K	3 (13)
E545K	3 (13)
H1047X	10 (44)
Other	3 (13)
Treatment cycles before	
1	7 (35)
2	6 (30)
3	4 (20)
4	3 (15)
Hyperglycemia during 3 days before introduction of alpelisib	11 (47.8)
Days from the last progression to the introduction of alpelisib, median (IQR)	25 (18-32)
Follow-up (days), median (IQR)	99 (56-154)

Data are presented as number (percentage) of patients if not state otherwise. Abbreviations: IQR, interquartile range; HbA1c, glycated hemoglobin A1c; ECOG, Eastern Cooperative Oncology Group; PIK3CA, phosphatidylinositol 3-kinase catalytic subunit alpha; hyperglycemia > 6.1 mmol/l (>110 mg/dL).

**Table 2.** Median hyperglycemia-free survival and prevalence of hyperglycemia relative to the introduction of alpelisib (*n* = 23).

	Value	[95% CI]
Median hyperglycemia-free survival (days)		
Any-grade	1	[1; 2]
Grade 2-4	6	[3; 44]
Grade 3-4	n.r.	[50; n.r.]
		
Prevalence of any-grade hyperglycemia		
During the 3 days before	11 (47.8)	[26.8; 69.4]
On the last day before	5 (21.7)	[7.5; 43.7]
During the first 7 days	21 (91.3)	[72.0; 98.9]
During days 8-14^t^	21 (91.3)	[72.0; 98.9]
During the first 14 days	22 (95.7)	[79.0; 99.2]
Prevalence of grade 2-4 hyperglycemia		
During the first 7 days	12 (52.2)	[30.6; 73.2]
During days 8-14	11 (47.8)	[26.8; 69.4]
During the first 14 days	14 (60.9)	[38.5; 80.3]
Prevalence of 3-4 hyperglycemia		
During the first 7 days	4 (17.4)	[5.0; 38.8]
During days 8-14	3 (13.0)	[2.8; 33.6]
During the first 14 days	4 (17.4)	[5.0; 38.8]
		
Median number of days with hyperglycemia during the first 14 days, median (IQR)		
Any grade	11 (7-13)	[8.7; 13.3]
Grade 2-4	2 (0-6)	[0; 4.3]

Data are presented as number (percentage) of patients if not state otherwise. Abbreviations: n.r., not reached; IQR, interquartile range; any grade hyperglycemia > 6.1 mmol/L (>110 mg/dL); grade 2-4 hyperglycemia > 8.9 mmol/L (>160 mg/dL); and grade 3-4 hyperglycemia > 13.9 mmol/L (>250 mg/dL).

### Occurrence and consequences of hyperglycemia

**Figure 2.**
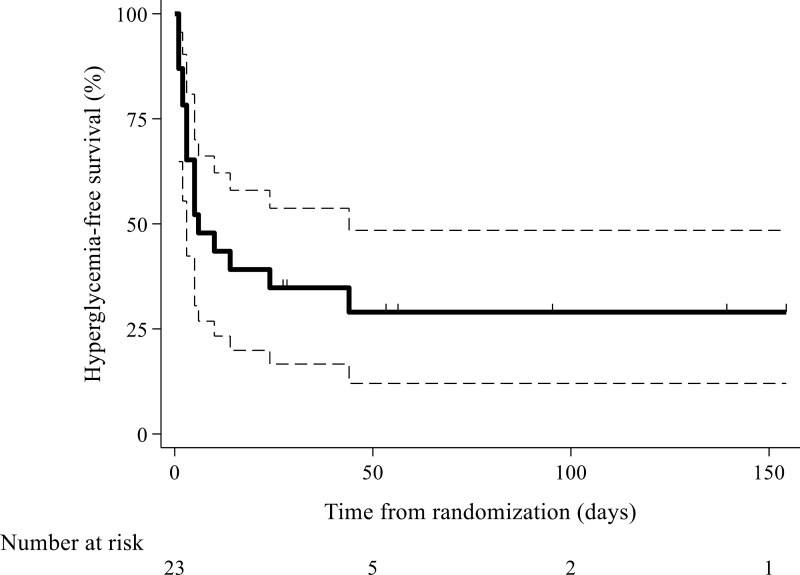
Hyperglycemia-free survival or time to the first hyperglycemia of grade 2 or 3 or 4; dotted lines represent 95% CI for survival curve (*n* = 23).

**Figure 3.**
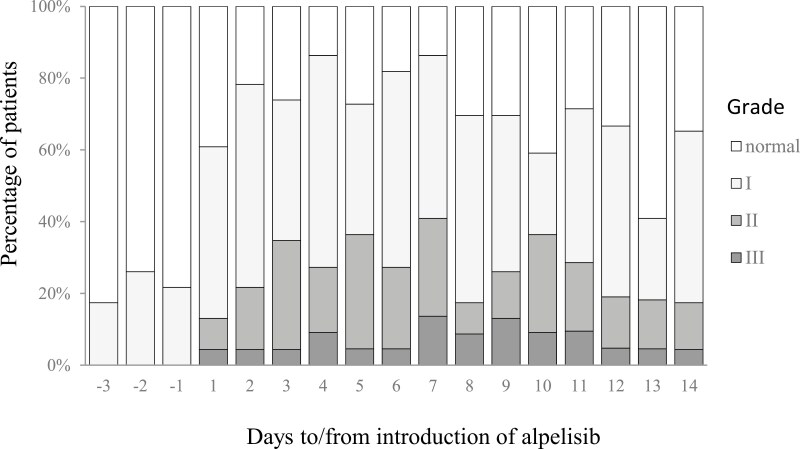
Hyperglycemia grade before breakfast by days to/from the introduction of alpelisib; white cells, no hyperglycemia; light gray, grade 1 [>6.1-8.9 mmol/L (>110-160 mg/dL)], darker gray, grade 2 [>8.9-13.9 mmol/L (>160-250 mg/dL)], darkest gray, grade 3 [>13.9-27.8 mmol/L (>250-500 mg/dL)] (*n* = 23); missing data: 1 (4%) on days 4, 5, 6, 7, 10, 13, and 2 (9%) on days 11 and 12.

Median grade 2-4 hyperglycemia-free survival was 6 days (95% CI, 3; 44 days), and median grade 3-4 hyperglycemia-free survival was not reached (Table 2, Figure 2). During the first 7 days after alpelisib initiation, any-grade hyperglycemia occurred in 21 (91.3%; 95% CI, 72.0; 98.9%) patients (Table 2, Figure 3). Prevalence of grade 2-4 hyperglycemia during the first 14 days of treatment with alpelisib was 14 (60.9%; 95% CI, 38.5; 80.3%), and prevalence of grade 3-4 (17.4%; 95% CI, 5.0; 38.8%) (Table 2, Supplementary Table S2). Median number of days patients spent in hyperglycemia was 11 (IQR 7-13; 95% CI, 8.7; 13.3). Median HbA1c at enrollment was 5.6 (IQR 5.2-5.9). After 14 days of treatment with alpelisib and fulvestrant, median HbA1c was 5.8 (IQR 5.4-6.6), what was absolute increase of 0.3 (IQR 0.1-0.6) and increase relative to the pretreatment HbA1c value, of 5.3 percentage points (IQR 2.0%-11.5%). Treatment was discontinued because of hyperglycemia in 1 (4.3%) patient, temporarily paused in 2 (8.7%), and alpelisib dose was lowered in 5 (21.7%) patients.

### Changes of fasting blood glucose concentrations

**Figure 4.**
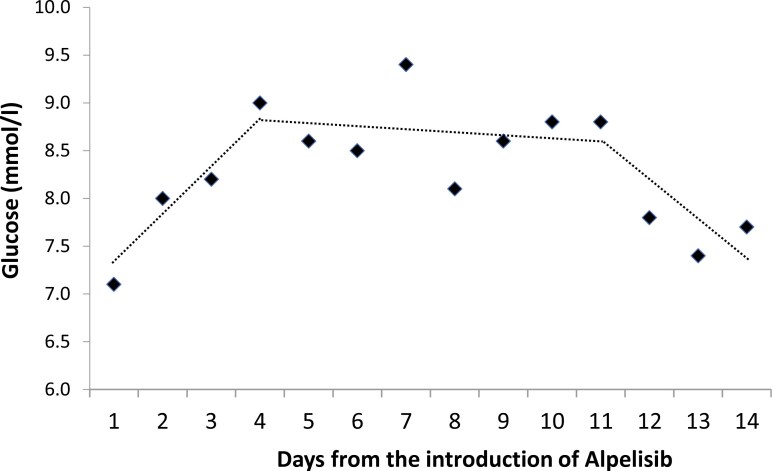
Mean glucose by days from the introduction of alpelisib; dotted trend lines represent joinpoint regression lines; missing data: 1 (4%) on days 4, 5, 6, 7, 10, 13, and 2 (9%) on days 11 and 12.

Based on the lowest BIC, the model that best fit the empirical data was the model with 2 join points, that is, 3 trends of changes in FBG concentrations during the first 14 days after the alpelisib initiation (Figure 4). The first join point was observed at day 4 (95% CI 3rd; 6th day) and the second at day 11 (95% CI 7th; 12th day). In the first period, from the introduction of alpelisib to the day 4, glucose concentration increased at a statistically significant DPC rate of 6.7% (95% CI, 3.5%: 13.6%). In the second period, from the day 5 to day 10, the trend in FBG change was not statistically significantly different from zero. Its DPC was −0.5% (95% CI, −1.8%; 1.3%). The difference in the slope of the regression line in the first and second periods was statistically significant (beta = −0.07; *P* =.043; FDR < 5%). In the third period, from days 11 to 14, the decrease in FBG concentration was DPC −5.0% (95% CI, −11.0%; −2%) and was again statistically significantly different from zero.

## Discussion

The exploratory interim analysis we performed on the pooled sample of first 23 patients enrolled in the ITACA trial demonstrated a rapid onset of hyperglycemia following alpelisib initiation, with most patients experiencing this adverse event within the first week of treatment. Frequency of any grade hyperglycemia that we observed (95.7%) was markedly higher than the one observed in SOLAR-1 trial (63.7%),^8^ the ones in 2 retrospective, real-world studies conducted by Cheung et al. (64.5%)^14^ and Alaklabi et al. (59.3%)^15^ as well as the ones in the systematic review and meta-analysis of alpelisib toxicity conducted by Shields at al. (60%).^16^

The most likely background for higher-than-expected incidence of hyperglycemia lies in the fact that we have measured occurrence and dynamics of hyperglycemia more often than in above stated trials. Also, the population in our study could be more “real life” and more like the average patient with metastatic, luminal type BC in the western world today. According to the results of a recently published study,^17^ conducted in 12 European countries, almost half of the patients were obese or overweight (48.1%). The obesity is a well-known risk factor for diabetes whose prevalence in 20-79 years olds in 2021 was estimated to be 10.5% (536.6 million people), rising to 12.2% (783.2 million) in 2045.^18^ Consistent with this information, in our study, 47.8% of the population during the 3 days prestudy period was observed with Grade 1 hyperglycemia, defining our population as high-risk for developing clinically relevant alpelisib-induced hyperglycemia. Bearing this information in mind, it is understandable why it would affect the occurrence of hyperglycemia while prescribing alpelisib, which is the most common cause of discontinuation of therapy according to SOLAR-1 trial (6.3%).^19^

The frequency of grade 2-4 in this exploratory interim analysis was somewhat higher than in SOLAR I, 60.9% in this analysis and 52.4% in SOLAR-1.^10^ Finally, in SOLAR-1 trial, 36.6% of patients experienced grade 3-4 hyperglycemia, compared with 17.4% of patients in this exploratory interim analysis, 19% in Cheung et al.,^14^ 25.9% in study conducted by Alaklabi et al.^15^ or 21% in retrospective cohort study by Sarfraz et al.^20^ Most likely reason for lower Grade 3 or Grade 4 hyperglycemia detected in our trial was due to early hyperglycemia detection and management applied. The prevalence of any grade hyperglycemia that we observed was more like the one found in the standard care arm of Shen et al. study (80.3%),^20^ but the median time to onset of hyperglycemia was markedly shorter in this exploratory interim analysis of the ITACA trial: 6 days for grade 2-4 hyperglycemia compared with 16 days in Shen et al. study.^20^ This is the most important finding of our analysis and have the potential of practice changing. More often and earlier glucose measurements should be introduced in everyday clinical practice to diagnose and manage hyperglycemia better.

In this exploratory interim analysis as well, respecting the low number of subjects, hyperglycemia was the most common cause of treatment discontinuation, and treatment with alpelisib was discontinued in 4.3% of patients, thus like SOLAR-1^8^ and to Shen et al. study (4.5%),^20^ but smaller than in real-world study by Cheung et al. (14.5%).^14^

### Study limitations

The main limitation of this interim analysis is the small sample size and its exploratory nature. Results may change as more data become available.

## Conclusion

Despite its rather small sample size, the results of our exploratory interim analysis indicate that precise, timely, and regular measurement of blood sugar levels may be necessary to detect the occurrence of hyperglycemia in our patients on time. Given that alpelisib is a drug with a short half-life (8-9 hours) and that hyperglycemia induced by its use is reversible and relatively easy to monitor and treat, timely recognition of alpelisib-induced hyperglycemia increases the possibility of proper management and therefore longer term treatment with alpelisib, thereby delaying the transition to the next line of treatment. This exploratory interim analysis demonstrated the rapid onset and high incidence of hyperglycemia in patients receiving alpelisib, even with the ITACA trial’s intervention. Proactive monitoring and early management of hyperglycemia may be crucial in this patient population. Patients directed for a therapy with alpelisib should be monitored more often and earlier, on daily bases for a first week of therapy with glucose measurements in everyday clinical practice to manage hyperglycemia better. If possible, these patients should be monitored using continuous glucose monitoring systems, devices which could detect glucose disturbances as early as possible. Therefore, we recommend daily glucose monitoring during the first week of alpelisib therapy, along with prompt intervention strategies, as this approach may improve treatment adherence and persistence, reduce adverse events, and potentially enhance the long-term efficacy of alpelisib. This exploratory interim analysis focuses on pooled data, and detailed intergroup comparisons will be reported in the final analysis.

## Supplementary Material

oyaf023_suppl_Supplementary_Tables_1-2

## Data Availability

The data and Stata code are available from the corresponding author upon request.
