# Supplementary material for: Dynamics of hyperglycemia of patients treated with alpelisib: exploratory interim analysis of ITACA trial
Source: Oncologist. 2025 Mar 27;30(3):oyaf023. doi: 10.1093/oncolo/oyaf023 (PMC11950915; doi:10.1093/oncolo/oyaf023)
Supplement: oyaf023_suppl_Supplementary_Tables_1-2 [file oyaf023_suppl_supplementary_tables_1-2.docx]

# SUPPLEMENTARY MATERIALS

**Supplementary Table 1.** Glucose before breakfast by patient, and day to/from the introduction of alpelisib (n = 23)

| ID | Days  to H | Folow-up | Days to/from introduction of alpelisib | | | | | | | | | | | | | | | | |
| --- | --- | --- | --- | --- | --- | --- | --- | --- | --- | --- | --- | --- | --- | --- | --- | --- | --- | --- | --- |
|  |  |  | -3 | -2 | -1 | 1 | 2 | 3 | 4 | 5 | 6 | 7 | 8 | 9 | 10 | 11 | 12 | 13 | 14 |
| 103 | 1 | 134 | 6.1 | 6.8 | 5.8 | 9.6 | 10.8 | 11.9 | 18.0 | 8.3 | 6.7 | 6.4 | 6.6 | 6.1 | 8.1 | 6.9 | 8.3 | 7.6 | 10.2 |
| 108 | 1 | 99 | 6.5 | 9.9 | 10.0 | 18.7 | 21.7 | 23.7 | 23.1 | 22.1 | 21.3 | 20.0 | 23.0 | 22.0 | 24.7 | 23.0 | 22.4 | 20.5 | 22.9 |
| 112 | 1 | 112 | 6.0 | 7.5 | 6.9 | 9.7 | 10.7 | 10.3 | 10.6 | 13.9 | 13.6 | 17.9 | 16.8 | 21.6 | 21.4 | 20.5 | 11.0 | 8.0 | 7.1 |
| 105 | 2 | 196 | 5.5 | 6.6 | 5.9 | 7.4 | 11.0 | 9.9 | 10.3 | 11.0 | 11.1 | 15.8 | 11.8 | 15.2 | 9.4 | 10.2 |  |  | 8.6 |
| 200 | 2 | 28 | 6.1 | 5.4 | 6.1 | 7.0 | 9.1 | 9.8 | 9.9 | 10.9 | 10.6 | 11.7 | 7.5 | 7.2 | 6.0 | 7.5 | 5.5 | 9.1 | 8.9 |
| 101 | 3 | 224 | 6.6 | 6.1 | 6.2 | 7.2 | 7.3 | 9.4 | 11.2 | 9.3 | 7.1 | 7.2 | 7.3 | 9.4 | 11.2 | 9.3 | 7.1 | 5.7 | 6.6 |
| 503 | 3 | 84 | 5.1 | 5.2 | 5.1 | 4.4 | 7.6 | 9.0 | 8.1 | 7.3 | 9.1 | 9.2 | 6.8 | 6.2 | 5.8 | 6.3 | 5.4 | 5.9 | 5.2 |
| 504 | 3 | 56 | 6.7 | 5.8 | 5.9 | 6.3 | 7.7 | 9.8 | 7.6 | 8.0 | 7.9 | 12.9 | 9.8 | 11.3 | 9.7 | 10.2 | 9.8 | 9.8 | 9.1 |
| 104 | 5 | 308 | 5.7 | 5.6 | 7.8 | 7.5 | 6.6 | 6.5 | 7.8 | 9.2 | 8.7 | 7.8 | 7.6 | 8.7 | 9.4 | 13.1 | 8.1 | 13.7 | 7.9 |
| 501 | 5 | 112 | 7.2 | 5.2 | 5.1 | 7.3 | 8.1 | 4.8 | 7.9 | 9.5 | 8.2 | 9.8 | 7.8 | 7.2 | 8.8 | 7.8 | 6.7 | 5.9 | 6.0 |
| 505 | 5 | 14 | 5.9 | 4.8 | 5.2 | 6.2 | 7.1 | 6.8 | 8.3 | 9.6 | 7.6 | 9.1 | 7.6 | 7.4 | 3.4 | 7.2 | 6.3 | 5.8 | 7.9 |
| 502 | 6 | 112 | 5.0 | 5.8 | 7.5 | 6.4 | 6.5 | 6.8 | 8.9 | 7.6 | 10.0 | 9.8 | 7.6 | 9.6 | 9.6 | 8.3 | 11.4 | 7.3 | 7.8 |
| 106 | 10 | 161 | 4.9 | 6.7 | 5.4 | 6.7 | 7.7 | 7.1 | 6.7 | 5.8 | 7.7 | 7.7 | 5.8 | 6.2 | 9.7 | 4.9 | 5.5 | 5.8 | 4.8 |
| 100 | 14 | 63 | 4.7 | 6.9 | 5.7 | 5.6 | 6.4 | 8.1 | 6.9 | 6.4 | 7.6 | 6.1 | 5.6 | 6.9 | 6.1 | 7.4 | 7.0 | 7.5 | 9.6 |
| 113 | 24 | 98 | 5.8 | 5.0 | 5.8 | 6.1 | 7.4 | 7.1 | 7.8 |  |  |  | 7.8 | 6.3 | 6.4 | 5.6 | 5.3 | 5.4 | 6.4 |
| 202 | 27 | 27 | 5.0 | 5.3 | 5.3 | 6.7 | 6.9 | 6.3 | 6.3 | 6.7 | 6.3 | 7.4 | 6.1 | 5.2 | 6.0 | 5.8 | 7.6 | 5.8 | 5.9 |
| 201 | 28 | 28 | 4.9 | 5.4 | 5.4 | 5.8 | 6.5 | 7.9 |  | 8.6 | 8.3 | 8.0 | 5.4 | 6.1 | 6.5 | 7.4 | 6.2 | 5.8 | 6.4 |
| 109 | 44 | 184 | 5.3 | 5.0 | 5.7 | 7.8 | 6.2 | 6.8 | 6.9 | 7.1 | 6.6 | 7.8 | 6.7 | 7.3 | 7.9 | 6.6 | 7.8 | 6.9 | 7.3 |
| 102 | 53 | 53 | 5.6 | 5.5 | 5.2 | 6.1 | 5.7 | 5.7 | 8.8 | 6.0 | 6.4 | 6.3 | 5.9 | 5.8 |  |  |  | 5.4 | 6.3 |
| 500 | 56 | 56 | 5.0 | 4.6 | 4.5 | 5.2 | 5.9 | 5.5 | 5.1 | 5.3 | 5.0 | 6.2 | 6.2 | 5.8 | 5.6 | 5.8 | 6.3 | 5.7 | 5.7 |
| 110 | 96 | 95 | 6.1 | 5.0 | 4.8 | 5.4 | 5.9 | 5.6 | 5.9 | 5.7 | 5.8 | 5.7 | 6.0 | 5.8 | 6.1 | 5.4 | 5.8 | 5.7 | 5.7 |
| 107 | 139 | 139 | 5.4 | 4.7 | 4.7 | 5.0 | 6.0 | 6.1 | 6.7 | 5.4 | 5.4 | 7.7 | 4.9 | 5.3 | 5.6 | 5.1 | 4.9 | 4.8 | 5.9 |
| 111 | 154 | 154 | 4.6 | 4.1 | 4.4 | 4.3 | 4.2 | 4.8 | 5.0 | 5.1 | 5.9 | 6.0 | 6.5 | 6.3 | 6.0 |  | 4.9 | 4.9 | 4.9 |

Abbreviations: ID, patient ID; H, hyperglycemia; white cells, no hyperglycemia; light gray, grade 1 [> 6.1 mmol/l (>110 mg/dl)], darker gray, grade 2 [> 8.9-13.9 mmol/l (> 160-250 mg/dl)], darkest grey, grade 3 [> 13.9-27.8 mmol/l (> 250-500 mg/dl)] hyperglycemia; empty cell, missing data

**Supplementary Table 2.** Hyperglycemia grade by patient, and day to/from the introduction of alpelisib (n = 23)

| Patient | Days  to H | Days to/from introduction of alpelisib | | | | | | | | | | | | | | | | |
| --- | --- | --- | --- | --- | --- | --- | --- | --- | --- | --- | --- | --- | --- | --- | --- | --- | --- | --- |
| ID |  | -3 | -2 | -1 | 1 | 2 | 3 | 4 | 5 | 6 | 7 | 8 | 9 | 10 | 11 | 12 | 13 | 14 |
| 103 | 1 | 0 | 1 | 0 | 2 | 2 | 2 | 3 | 1 | 1 | 1 | 1 | 0 | 1 | 1 | 1 | 1 | 2 |
| 108 | 1 | 1 | 1 | 1 | 3 | 3 | 3 | 3 | 3 | 3 | 3 | 3 | 3 | 3 | 3 | 3 | 3 | 3 |
| 112 | 1 | 0 | 1 | 1 | 2 | 2 | 2 | 2 | 2 | 2 | 3 | 3 | 3 | 3 | 3 | 2 | 1 | 1 |
| 105 | 2 | 0 | 1 | 0 | 1 | 2 | 2 | 2 | 2 | 2 | 3 | 2 | 3 | 2 | 2 |  |  | 1 |
| 200 | 2 | 0 | 0 | 0 | 1 | 2 | 2 | 2 | 2 | 2 | 2 | 1 | 1 | 0 | 1 | 0 | 2 | 1 |
| 101 | 3 | 1 | 0 | 1 | 1 | 1 | 2 | 2 | 2 | 1 | 1 | 1 | 2 | 2 | 2 | 1 | 0 | 1 |
| 503 | 3 | 0 | 0 | 0 | 0 | 1 | 2 | 1 | 1 | 2 | 2 | 1 | 1 | 0 | 1 | 0 | 0 | 0 |
| 504 | 3 | 1 | 0 | 0 | 1 | 1 | 2 | 1 | 1 | 1 | 2 | 2 | 2 | 2 | 2 | 2 | 2 | 2 |
| 104 | 5 | 0 | 0 | 1 | 1 | 1 | 1 | 1 | 2 | 1 | 1 | 1 | 1 | 2 | 2 | 1 | 2 | 1 |
| 501 | 5 | 1 | 0 | 0 | 1 | 1 | 0 | 1 | 2 | 1 | 2 | 1 | 1 | 1 | 1 | 1 | 0 | 0 |
| 505 | 5 | 0 | 0 | 0 | 1 | 1 | 1 | 1 | 2 | 1 | 2 | 1 | 1 | 0 | 1 | 1 | 0 | 1 |
| 502 | 6 | 0 | 0 | 1 | 1 | 1 | 1 | 1 | 1 | 2 | 2 | 1 | 2 | 2 | 1 | 2 | 1 | 1 |
| 106 | 10 | 0 | 1 | 0 | 1 | 1 | 1 | 1 | 0 | 1 | 1 | 0 | 1 | 2 | 0 | 0 | 0 | 0 |
| 100 | 14 | 0 | 1 | 0 | 0 | 1 | 1 | 1 | 1 | 1 | 0 | 0 | 1 | 0 | 1 | 1 | 1 | 2 |
| 113 | 24 | 0 | 0 | 0 | 0 | 1 | 1 | 1 |  |  |  | 1 | 1 | 1 | 0 | 0 | 0 | 1 |
| 202 | 27 | 0 | 0 | 0 | 1 | 1 | 1 | 1 | 1 | 1 | 1 | 0 | 0 | 0 | 0 | 1 | 0 | 0 |
| 201 | 28 | 0 | 0 | 0 | 0 | 1 | 1 |  | 1 | 1 | 1 | 0 | 0 | 1 | 1 | 1 | 0 | 1 |
| 109 | 44 | 0 | 0 | 0 | 1 | 1 | 1 | 1 | 1 | 1 | 1 | 1 | 1 | 1 | 1 | 1 | 1 | 1 |
| 102 | 53 | 0 | 0 | 0 | 0 | 0 | 0 | 1 | 0 | 1 | 1 | 0 | 0 |  |  |  | 0 | 1 |
| 500 | 56 | 0 | 0 | 0 | 0 | 0 | 0 | 0 | 0 | 0 | 1 | 1 | 0 | 0 | 0 | 1 | 0 | 0 |
| 110 | 96 | 0 | 0 | 0 | 0 | 0 | 0 | 0 | 0 | 0 | 0 | 0 | 0 | 0 | 0 | 0 | 0 | 0 |
| 107 | 139 | 0 | 0 | 0 | 0 | 0 | 0 | 1 | 0 | 0 | 1 | 0 | 0 | 0 | 0 | 0 | 0 | 0 |
| 111 | 154 | 0 | 0 | 0 | 0 | 0 | 0 | 0 | 0 | 0 | 0 | 1 | 1 | 0 |  | 0 | 0 | 0 |

Abbreviations: ID, patient ID; H, hyperglycemia; white cells, no hyperglycemia; light gray, grade 1 [> 6.1 mmol/l (>110 mg/dl)], darker gray, grade 2 [> 8.9-13.9 mmol/l (> 160-250 mg/dl)], darkest grey, grade 3 [> 13.9-27.8 mmol/l (> 250-500 mg/dl)] hyperglycemia; empty cell, missing data
